# Amplified B Lymphocyte CD40 Signaling Drives Regulatory B10 Cell Expansion in Mice

**DOI:** 10.1371/journal.pone.0022464

**Published:** 2011-07-25

**Authors:** Jonathan C. Poe, Susan H. Smith, Karen M. Haas, Koichi Yanaba, Takeshi Tsubata, Takashi Matsushita, Thomas F. Tedder

**Affiliations:** 1 Department of Immunology, Duke University Medical Center, Durham, North Carolina, United States of America; 2 Laboratory of Immunology, Graduate School of Biomedical Sciences, Tokyo Medical and Dental University, Tokyo, Japan; University of Miami, United States of America

## Abstract

**Background:**

Aberrant CD40 ligand (CD154) expression occurs on both T cells and B cells in human lupus patients, which is suggested to enhance B cell CD40 signaling and play a role in disease pathogenesis. Transgenic mice expressing CD154 by their B cells (CD154^TG^) have an expanded spleen B cell pool and produce autoantibodies (autoAbs). CD22 deficient (CD22^−/−^) mice also produce autoAbs, and importantly, their B cells are hyper-proliferative following CD40 stimulation *ex vivo*. Combining these 2 genetic alterations in CD154^TG^CD22^−/−^ mice was thereby predicted to intensify CD40 signaling and autoimmune disease due to autoreactive B cell expansion and/or activation.

**Methodology/Principal Findings:**

CD154^TG^CD22^−/−^ mice were assessed for their humoral immune responses and for changes in their endogenous lymphocyte subsets. Remarkably, CD154^TG^CD22^−/−^ mice were not autoimmune, but instead generated minimal IgG responses against both self and foreign antigens. This paucity in IgG isotype switching occurred despite an expanded spleen B cell pool, higher serum IgM levels, and augmented *ex vivo* B cell proliferation. Impaired IgG responses in CD154^TG^CD22^−/−^ mice were explained by a 16-fold expansion of functional, mature IL-10-competent regulatory spleen B cells (B10 cells: 26.7×10^6^±6 in CD154^TG^CD22^−/−^ mice; 1.7×10^6^±0.4 in wild type mice, p<0.01), and an 11-fold expansion of B10 cells combined with their *ex vivo*-matured progenitors (B10+B10pro cells: 66×10^6^±3 in CD154^TG^CD22^−/−^ mice; 6.1×10^6^±2 in wild type mice, p<0.01) that represented 39% of all spleen B cells.

**Conclusions/Significance:**

These results demonstrate for the first time that the IL-10-producing B10 B cell subset has the capacity to suppress IgG humoral immune responses against both foreign and self antigens. Thereby, therapeutic agents that drive regulatory B10 cell expansion *in vivo* may inhibit pathogenic IgG autoAb production in humans.

## Introduction

Interactions between CD40 expressed by B cells and its ligand CD154, expressed by antigen (Ag)-activated CD4^+^ helper T cells, promotes BCR-induced B cell proliferation and survival, which is essential for isotype switching and germinal center (GC) formation [1], [2], [3], [4], [5], [6]. Interrupting CD40 and CD154 interactions prevents the development of both T cell-dependent (TD) humoral immune responses and cell-mediated immune responses [7]. Agonistic CD40 mAbs are also potent immune adjuvants for both short-lived humoral-immunity to T cell-independent Ags [8], [9] and cellular immune responses to viruses and tumors [10], [11], [12]. However, CD40 agonists given during TD immune responses actually ablate GC formation, induce a pattern of extrafollicular B cell differentiation in the spleen and lymph nodes, prematurely terminate humoral immune responses, block the generation of B cell memory, and prevent the generation of long-lived bone marrow plasma cells [13]. Consistent with this, ectopic CD154 expression by B cells in transgenic mice (CD154^TG^) terminates germinal center responses prematurely and leads to augmented plasma cell production in T cell areas [14], [15]. Expression of the CD154 transgene in these mice is driven by immunoglobulin (Ig) gene promoter and enhancer elements, resulting in B cell-specific expression [14], [15]. B cell CD154 expression has a precedent in human disease, as it is expressed by both T cells and B cells in systemic lupus erythematosus (SLE) patients and in a mouse model of lupus [16], [17], [18], with ectopic B cell expression of CD154 in aged hemizygous CD154^TG^ mice leading to intestinal inflammation [19] or SLE-like autoimmunity including anti-DNA autoAbs and glomerulonephritis [20]. While a certain level of B cell CD40 signaling can exacerbate the development or severity of autoimmune disease, these studies collectively suggest that the fate of Ag-specific B cells is dramatically altered by the extent of CD40 ligation, with heightened CD40 signaling potentially representing a physiological means to limit the duration and intensity of immune responses.

CD22 negatively regulates transmembrane signals in B cells through association with the potent intracellular phosphatases SHP-1 and SHIP [21], [22], [23], [24]. B cells from CD22^−/−^ mice are markedly hyper-responsive to CD40 signals, whereby their *ex vivo* stimulation with agonistic CD40 mAb induces a much greater degree of proliferation relative to wild type (WT) B cells [25]. As such, potent *in vivo* signals provided by constitutive CD40 signaling combined with CD22 deficiency may alter the duration and intensity of immune responses, size of the autoreactive B cell pool, and autoAb production levels. To test this, CD22^−/−^ mice homozygous for the CD154 transgene (CD154^TG^CD22^−/−^) were generated. Remarkably, the defining *in vivo* characteristic of CD154^TG^CD22^−/−^ mice was a dramatic expansion in regulatory B10 cells that were competent to express IL-10 [26], [27], and meager IgG production against both foreign and self Ags. Thus, enhancing CD40 signaling limited the duration and intensity of humoral immune responses likely by driving the expansion of B10 cells, a B cell subset that is also found in humans [28]. Inducing such an expansion of B10 cells may be particularly therapeutic in autoimmune syndromes such as SLE where aberrant CD154 expression contributes to inflammation and the generation of pathogenic isotype-switched B cells.

## Methods

### Ethics statement

All animal studies and procedures were approved by the Duke University Institutional Animal Care and Use Committee (approved IACUC protocol #A008-08-01; Duke University PHS Animal Welfare Assurance No. A3195-01).

### Mice

CD22^−/−^ mice, backcrossed with C57BL/6J mice (Jackson Laboratories, Bar Harbor, ME) for ≥8 generations were previously described [25]. CD154 transgenic mice [20] were crossed to homozygosity and referred to as CD154^TG^ mice. Double mutant mice were generated by interbreeding the F1 offspring of CD22^−/−^ and CD154^TG^ mice, with CD154^TG^CD22^−/−^ mice maintained as homozygous at both genetic loci by sibling matings. C57BL/6 WT control mice were purchased from either The Jackson Laboratory (Bar Harbor, ME) or NCI Frederick (Bethesda, MD). Bcl-xL transgenic mice [29] were a kind of Dr. Michael Farrar (University of Minnesota, Minneapolis, MN). Unless otherwise indicated, all mice used in these studies were between 8 and 14 weeks of age. Mice were housed in a specific pathogen-free barrier facility.

### Tissue harvest, flow cytometry and Abs

Single-cell suspensions were isolated from spleen, BM, peripheral LNs, and the peritoneal cavity. Spleen and BM RBCs were depleted using ammonium chloride-Tris lysis buffer. Blood was obtained by retroorbital puncture. Leukocytes (0.5–1×10^6^) were stained at 4°C using predetermined optimal concentrations of Abs for 30 min, then analyzed by flow cytometry. Blood RBCs were lysed following surface staining using BD FACS™ Lysing Solution (BD Biosciences). Cells were analyzed on a FACSCanto II flow cytometer (BD Biosciences). Abs against surface or intracellular mouse proteins were as follows: B220 (RA3-6B2), CD21/35 (7G6), CD24 (M1/69), CD25 (PC61), GL-7 (Ly-77), CD154 (MR1), CD23 (B3B4), CD44 (IM7), CD1d (1B1), CD90.2 (53-2.1), all from BD Pharmingen; IgM (11/41), B220 (RA3-6B2), CD21/35 (eBio8D9), CD23 (B3B4), CD93 (AA4.1), CD1d (1B1), CD5 (53-7.3), CD62L (MEL-14), Foxp3 (FKJ-16s), IL-10 (JES5-16E3), MHC class II (I-A^b^ clone MC/114), all from eBioscience Inc. (San Diego, CA); goat anti-mouse IgM or IgD (Southern Biotechnology Associates, Inc.) To detect intracellular IL-10, a BD Cytofix/Cytoperm Kit was used. To detect intracellular Foxp3, a Foxp3 Staining Buffer Set was used (eBioscience Inc.).

### B cell isolation, proliferation and survival assays

Single cell splenocyte suspensions isolated under aseptic conditions were enriched for B cells using a B Cell Isolation Kit (Miltenyi Biotech), yielding >95% B220^+^ cells. B cells (1×10^6^ cells/ml) were cultured in RPMI 1640 medium supplemented with 10% FCS, 10 mM HEPES, 55 µM 2-mercaptoethanol either alone or in the presence of F(ab′)_2_ goat anti-mouse IgM Ab (Cappel), or CD40 mAb (clone HM40-3, BD Pharmingen). Proliferation was measured by incorporation of [^3^H]-thymidine (1 µCi/well) added during the final 18 h of 72 h cultures, followed by scintillation counting. Alternatively, B cells were labeled with 1 µM CFSE before culture using a Vybrant CFDA SE Cell Tracer Kit (Molecular Probes) and analyzed for CFSE dilution by flow cytometry, with viable cells determined by 7-amino-actinomycin D (7-AAD) exclusion.

### Immunofluorescence microscopy

For visualization of CD1d^hi^ B cells *in situ*, 5 µm cryostat spleen sections were stained with FITC-conjugated B220 mAb and PE-conjugated CD1d mAb. Digital images were merged to identify CD1d^hi^ B cells within follicles and MZ regions. To visualize GL7^+^ B cells *in situ*, spleen sections were stained with FITC-conjugated GL7 mAb and PE-conjugated B220 mAb. Digital images were merged to identify GL7^+^ B cells within GCs or other follicle regions.

### Experimental autoimmune encephalomyelitis (EAE) experiments

Purified spleen B cells from CD154^TG^CD22^−/−^ donor mice were surface labeled with CD1d and CD5 mAbs, with CD1d^hi^CD5^+^ and CD1d^lo^CD5^−^ B cell populations isolated using a FACSVantage SE flow cytometer with purities of 95–98%. 1×10^6^ cells were either directly transferred i.v. into WT recipient mice, or were first cultured with agonistic CD40 mAb for 48 h with LPS added during the final 5 h of culture, then washed and transferred. One day after cell transfers, EAE was induced in recipient mice with clinical signs of EAE scored daily as described [30], [31].

### Immunizations

In some experiments, WT, CD22^−/−^, CD154^TG^, and CD154^TG^CD22^−/−^ mice were immunized i.p. with 100 µg DNP-KLH in CFA, and then boosted on day 21 with DNP-KLH in IFA. Serum Ag-specific Ig levels were determined by ELISA. Alternatively, the mice were immunized i.p. with 50 µg 4-hydroxy-3-nitrophenyl acetyl-conjugated chicken γ-globulin (NP_8_CGG) in alum, with spleens were harvested on day 10 and the splenocytes stained with GL-7 and B220 mAbs, with double-positive B cells identified by flow cytometry. In other experiments where adjuvant was excluded, WT mice were immunized i.p. with 100 µg DNP-KLH in PBS alone, and then boosted on day 21 with DNP-KLH in PBS alone.

### ELISAs and ELISPOT assays

Serum IgM and IgG levels and serum IgM and IgG autoAb levels were determined by ELISA. 96-well microtiter plates (Costar, Cambridge, MA) were coated overnight at 4°C with either 5 µg/ml goat anti-mouse Ig(H+L) Abs (BD Pharmingen), 2 µg/ml of calf thymus double- or single-stranded DNA (Sigma-Aldrich, St. Louis, MO), or 5 µg/ml calf thymus Type II-AS histone proteins (Sigma-Aldrich). Single stranded DNA was obtained by boiling calf thymus dsDNA in 1× SSC for 10 minutes, followed by immediate immersion in ice. Coated plates were blocked for 1 hr at RT with Tris-buffered saline containing 1% BSA and 2% gelatin, then incubated for 1 hr at RT with pre-determined concentrations of sera diluted in Tris-buffered saline containing 1% BSA. Bound Abs were detected with alkaline phosphatase-conjugated goat anti-mouse IgG or IgM Abs (Southern Biotechnology Associates, Birmingham, AL). Ig levels were determined by quantifying serum titers against known standards (purified mouse IgM or IgG, Southern Biotechnology Associates). Alkaline phosphatase activity was detected using *p*-nitrophenyl phosphate substrate (Sigma-Aldrich) at a wavelength of 405 nm.

DNP-specific Ab levels were determined by ELISA as described [32]. Briefly, sera from DNP-KLH-immunized mice were diluted 1∶1000 for analysis using ELISA plates coated overnight at 4°C with 100 µg/ml DNP-BSA (Calbiochem-Novabiochem Corp.). Bound Ag-specific IgM and IgG Abs were then detected with alkaline phosphatase-conjugated goat anti-mouse IgM or isotype-specific IgG Abs, as above. Ab-secreting cell frequencies were determined by ELISPOT assay as described [33]. Briefly, dilutions of spleen B cells were cultured in DNP-BSA-coated Multiscreen HTS IP plates (Millipore, Bedford, MA) for 5 hours. After extensive washing, the plates were incubated with alkaline phosphatase-conjugated goat anti-mouse IgG Ab, with the spots counted using a dissecting microscope.

### CD22 mAb treatment

The MB22-10 mAb and an isotype-matched control mAb (250 µg/mouse) were given to mice i.v. as previously described to deplete CD1d^hi^CD5^+^ B cells [34] and IL-10-competent B10 cells [31].

### Statistical analysis

All data are shown as means (±SEM), unless otherwise noted. The Student's t-test was used to determine the significance of differences between sample means.

## Results

### B cell phenotype and numbers in CD154^TG^CD22^−/−^ mice

**Figure 1:**
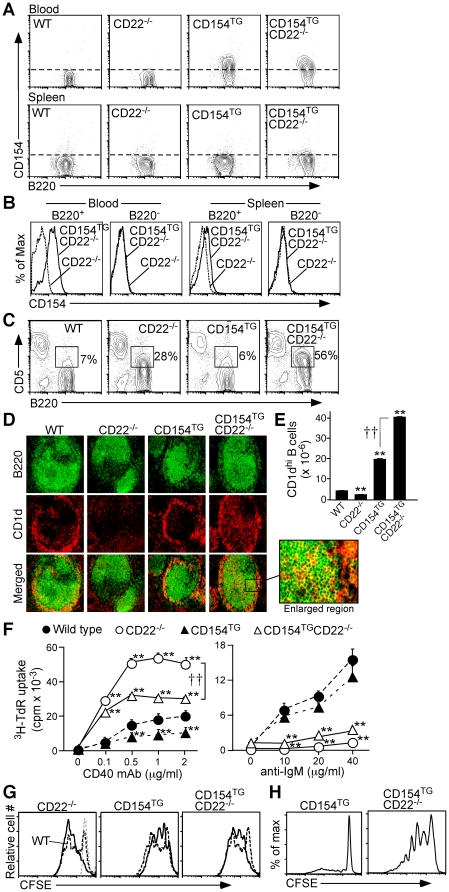
B cell development in CD154^TG^CD22^−/−^ and CD154^TG^ mice. (**A**) B cell CD154 expression in WT, CD22^−/−^, CD154^TG^ and CD154^TG^CD22^−/−^ mice. Blood and spleen B220^+^ B cells were assessed for CD154 expression by immunofluorescence staining with flow cytometry analysis. Dashed lines delineate borders between CD154^+^ and CD154^−^ cells as determined using WT lymphocytes. (**B**) Ectopic CD154 expression is B cell-restricted in CD154^TG^CD22^−/−^ mice. CD154 expression by blood and spleen B220^+^ and B220^−^ mononuclear cells was assessed by immunofluorescence staining with flow cytometry analysis. Results represent those obtained in 6 pairs of mice. (**C**) Increased CD5 expression by B cells from CD22^−/−^ and CD154^TG^CD22^−/−^ mice. Spleen B cells were assessed for CD5 expression by immunofluorescence staining. Gates indicate percentages of CD5^+^ B cells among total B220^+^ cells. (**D**) Spleen CD1d^hi^B220^+^ B cell localization. Tissue sections from WT, CD22^−/−^, CD154^TG^, and CD154^TG^CD22^−/−^ mice were stained with B220 (FITC, green) and CD1d (PE, red) mAbs. Merged images highlight CD1d^hi^B220^+^ cells (yellow). (A–C) Results are representative of ≥3 mice of each genotype. (**E**) Spleen CD1d^hi^B220^+^ B cell numbers in WT, CD22^−/−^, CD154^TG^ and CD154^TG^CD22^−/−^ mice as determined by immunofluorescence staining. Results represent the mean (±SEM) from ≥3 mice of each genotype. (**F**) CD154^TG^CD22^−/−^ B cells are hyper-responsive to CD40 signals. Spleen B cells from WT, CD22^−/−^, CD154^TG^, and CD154^TG^CD22^−/−^ mice were cultured with mitogenic CD40 mAb or anti-IgM Ab for 72 h, with [^3^H]-thymidine incorporation assessed during the final 18 h of culture. Values represent means (±SEM) of triplicate cultures. (E–F) Means significantly different from WT values are indicated by asterisks (**p<0.01), and between other genotypes by crosses (††p<0.01). (**G**) CD154^TG^CD22^−/−^ B cell proliferation in response to LPS stimulation. Cell division of CFSE-labeled B cells from WT, CD22^−/−^, CD154^TG^ and CD154^TG^CD22^−/−^ mice following LPS stimulation was quantified after 72 h in culture by flow cytometry. (F–G) Results are representative of 2 independent experiments with similar results. (**H**) Enhanced survival by CFSE-labeled B cells from CD154^TG^ and CD154^TG^CD22^−/−^ mice after 10 days in culture without mitogenic stimulation as assessed by flow cytometry. Results represent 3 independent experiments producing similar results.

The biological effects of CD22-deficiency are B cell intrinsic as CD22 expression is only expressed by B cells [25]. Furthermore, transgenic CD154 was specifically expressed by B cells in homozygous CD154^TG^ mice, but not on other lymphocytes or other hematopoietic cells in the blood or spleen (Figure 1A–B, data not shown) as previously documented for hemizygous CD154^TG^ mice [20]. To examine the combined effects of B cell intrinsic CD154 expression and CD22-deficiency, CD154^TG^CD22^−/−^ double mutant mice homozygous at both genetic loci were generated by intercrossing the F1 progeny of breedings between hemizygous CD154^TG^ mice [20] and CD22^−/−^ mice [25]. CD22^−/−^, CD154^TG^, and CD154^TG^CD22^−/−^ mice all developed and bred normally. A subpopulation of blood B cells of both CD154^TG^ (43±1%, n = 6) and CD154^TG^CD22^−/−^ (60±1%, n = 6) mice expressed detectable cell surface CD154 (Figure 1A), with intermediate CD154 expression by hemizygous mice (data not shown). Cell surface CD154 expression was much lower on spleen B cells, as previously reported for hemizygous CD154^TG^ mice [20], indicating CD154 internalization subsequent to CD40–CD154 engagement within the spleen.

CD154^TG^CD22^−/−^ spleen B cells exhibited the chronically-activated phenotype typical of CD22^−/−^ B cells [25], with increased CD5 expression (Figure 1C), as well as enhanced MHC class II and decreased cell surface IgM expression (data not shown). Spleen B220^+^IgM^+^ B cell numbers were increased by 2.5-fold in CD154^TG^CD22^−/−^ mice relative to WT mice, while a 3.6-fold increase was observed in CD154^TG^ mice (Table 1). Spleen Thy1.2^+^ T cell and Foxp3^+^CD25^+^CD4^+^ regulatory T cell (Treg) numbers did not differ significantly between WT, CD22^−/−^, CD154^TG^, and CD154^TG^CD22^−/−^ mice. However, memory CD4^+^ and CD8^+^ T cell numbers were significantly increased in CD154^TG^CD22^−/−^ and CD154^TG^ mice. Thus, the predominant effect of ectopic CD154 expression was the significant expansion of spleen B cells in CD22^−/−^ mice, coupled with expanded memory T cell populations.

**Table 1:** Lymphocyte subsets in WT, CD22^−/−^, CD154^TG^, and CD154^TG^CD22^−/−^ mice^a^.

		Lymphocyte number (×10^−6^)			
Tissue	Phenotype	WT	CD22^−/−^	CD154^TG^	CD154^TG^CD22^−/−^
BM:	B220^hi^IgM^hi^	1.5±0.3	0.4±0.2*	1.2±0.3	0.5±0.03*
	B220^hi^HSA^lo^	1.4±0.2	0.3±0.1**	1.4±0.3	0.3±0.04**
Blood:	B220^+^	2.7±1	0.9±0.2*	3.9±1	0.4±0.1**
Spleen:	B220^+^IgM^+^	68±9	46±8	250±40**	171±20**
	B10^b^	1.7±0.4	1.8±0.4	6.5±2**	26.7±6**
	B10+B10pro	6.1±2	5.4±0.8	24±4**	66±3**
	IgM^+^IgD^+^CD23^hi^	56±8	42±7	224±50*	127±20*
	IgM^hi^CD21^hi^	5.6±0.9	1.4±0.3**	24±4**	29±6*
	CD1d^hi^CD5^+^	1.8±0.4	2.3±0.8	4.2±0.3**	31±5*
	B220^lo^CD5^+^	2.2±0.5	1.7±0.4	9.2±3*	6.6±1*
	Thy1.2^+^	39±5	38±7	39±4	53±9
	CD4^+^Foxp3^+^CD25^+^	3.4±0.2	3.3±0.4	2.7±0.4	4.4±0.8
	CD4^+^ T naïve	15±4	18±5	11±2	8.5±4
	CD4^+^ T memory	6.9±0.8	5.8±0.8	9.7±0.6*	14±3*
	CD8^+^ T naïve	13±2	14±3	5.9±1*	2.0±0.6**
	CD8^+^ T memory	0.7±0.08	0.8±0.1	9.1±1**	15±3**
	IL-10^+^ T cells^c^	0.6±0.2	0.8±0.2	6.4±2.5*	8.1±2.2**
PLN:	B220^+^	4.6±0.8	1.9±0.2*	9.4±1*	4.4±2
	B10	0.01±0.005	0.003±0.001	0.06±0.01*	0.2±0.1*
MLN:	B10	0.02±0.01	0.02±0.003	0.3±0.06**	0.4±0.2*
PC:	B220^+^IgM^+^	0.7±0.1	0.3±0.1	2.9±0.6*	1.2±0.3
	B10	0.6±0.1	0.5±0.1	0.7±0.1	0.7±0.3
	B220^lo^CD5^+^	0.4±0.1	0.3±0.1	1.0±0.3	0.4±0.1
	B220^hi^IgD^hi^	0.5±0.1	0.2±0.1	2.4±0.5*	1.0±0.2

a Numbers represent mean (±SEM) values from ≥4 littermates of each genotype. Asterisks indicate values significantly different from WT mice (*p<0.05; **p<0.01). PLN, peripheral LN; MLN, mesenteric LN; PC, peritoneal cavity.

b B10 and B10pro cells were identified as in Figure 3.

c IL-10-competent T cells were identified following 5 h stimulation with PMA/ionomycin.

The CD1d^hi^B220^+^ marginal zone (MZ) B cell population was significantly reduced in CD22^−/−^ mice as assessed in frozen spleen sections (Figure 1D), as published [24], [34], [35]. Remarkably, the MZ B cell population was restored in CD154^TG^CD22^−/−^ mice, revealing that CD40 ligation overcomes the effects of CD22 deficiency on MZ maintenance. Nevertheless, CD1d^hi^B220^+^ cells were also observed within B cell follicles of all genotypes. CD1d^hi^ B cells were particularly abundant within the MZ and follicles of CD154^TG^CD22^−/−^ mice (highlighted in the enlarged region), paralleling their overall high numbers relative to the other mouse lines (Figure 1E).

CD22^−/−^ B cells proliferate much more extensively than WT B cells when cultured with agonistic CD40 mAb [25]. B cells from CD154^TG^ mice proliferated less than WT B cells following CD40 stimulation as quantified by [^3^H]-thymidine incorporation (Figure 1F), suggesting desensitization of the CD40 pathway in these mice. However, CD154^TG^CD22^−/−^ B cells proliferated more extensively than WT B cells following CD40 stimulation, indicating that the hyper-responsiveness caused by CD22 deficiency persists in these cells. Altered CD40 expression does not explain these results, as cell surface CD40 expression was equivalent on spleen B cells from WT, CD22^−/−^, CD154^TG^ and CD154^TG^CD22^−/−^ mice as determined by immunofluorescence staining with flow cytometry analysis (data not shown). Similar to CD40 ligation, proliferation of CD154^TG^CD22^−/−^ and CD22^−/−^ B cells in response to LPS stimulation was modestly enhanced relative to WT or CD154^TG^ B cells as quantified by CFSE dilution analysis (Figure 1G).

In contrast to CD40 stimulation, CD22^−/−^ B cells undergo apoptosis following extensive BCR ligation due to blocked cell cycle progression [25]. Likewise, proliferation of B cells from CD154^TG^CD22^−/−^ mice was profoundly deficient following BCR ligation with F(ab)′_2_ anti-IgM Ab and nearly identical to the defect of CD22^−/−^ B cells (Figure 1F). Without CD40 or BCR stimulation, background levels of B cell [^3^H]-thymidine incorporation were low, but were significantly higher (P<0.01) for B cells from CD154^TG^CD22^−/−^ and CD154^TG^ mice than for WT and CD22^−/−^ mice (data not shown). Consistent with this, purified B cells from both CD154^TG^ and CD154^TG^CD22^−/−^ mice remained viable when cultured for >10 days without any exogenous mitogenic stimulation, while CD154^TG^CD22^−/−^ B cells uniquely proliferated at significant levels as assessed by CFSE dilution (Figure 1H). Collectively, these data demonstrate that B cells in CD154^TG^CD22^−/−^ mice are capable of receiving CD40 and additional costimulatory signals that promote B cell activation, survival, and proliferation.

Importantly, transgenic CD154 engagement of CD40 in CD154^TG^ and CD154^TG^CD22^−/−^ mice appears to be primarily mediated through *cis* (same cell) interactions on B cells, since mixed bone marrow chimeras derived from WT and CD154^TG^ donors exhibit enhanced Ab production specifically from CD154^TG^ B cells [14], [15]. Also, activation markers on dendritic cells are not altered in CD154^TG^ mice relative to WT mice [14], [15]. In addition, despite being hyper-responsive to stimulation with agonistic CD40 mAb, CFSE-labeled CD22^−/−^ spleen B cells co-cultured with CD154^TG^CD22^−/−^ spleen B cells *ex vivo* did not exhibit enhanced survival or proliferation compared to CD22^−/−^ B cells cultured alone (data not shown). These observations strongly suggest that the phenotypic alterations observed in CD154^TG^ and CD154^TG^CD22^−/−^ mice are mediated by enhanced, autocrine CD40 signaling in the B cell lineage, and not through the activation of bystander cells in other lineages that also express CD40.

### Reduced IgG Ab and autoAb production in CD154^TG^CD22^−/−^ mice

**Figure 2:**
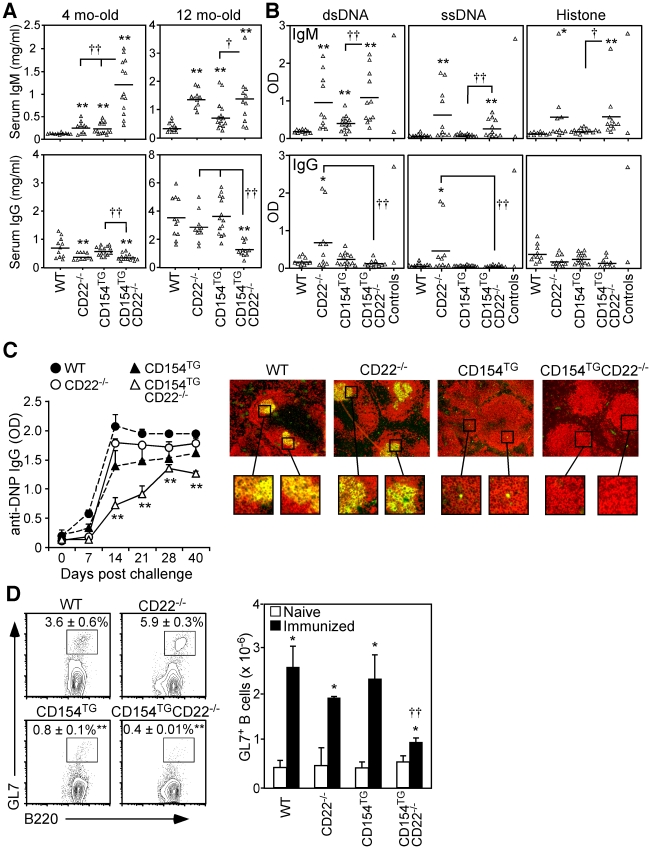
Impaired IgG and GC responses in CD154^TG^CD22^−/−^ mice. (**A**) Serum IgM and IgG levels of 4 and 12 mo-old WT, CD22^−/−^, CD154^TG^, and CD154^TG^CD22^−/−^ mice. Symbols represent serum concentrations for individual mice as determined by ELISA, with means indicated by horizontal bars. (**B**) Serum autoAbs reactive with dsDNA, ssDNA, or histone proteins in 12 mo-old mice. ELISA OD values for IgM (upper panels) and IgG (lower panels) autoAbs are shown for individual mice, with means indicated by horizontal bars. Sera from 2 mo-old WT C57BL/6 and 6 mo-old MLR*lpr* mice were used as negative and positive controls, respectively. (**C**) Impaired IgG responses to a TD Ag. WT (*n* = 3), CD22^−/−^ (*n* = 4), CD154^TG^ (*n* = 6), and CD154^TG^CD22^−/−^ (*n* = 8) mice were immunized with DNP-KLH in adjuvant on day 0, and boosted on day 21. The graph shows mean (±SEM) DNP-specific IgG levels as determined by ELISA. Images on the right represent immunofluorescence staining of frozen spleen sections from all genotypes harvested 7 days after the boost phase of DNP-KLH immunization. Merged images show the presence of B220^+^ B cells (red) and GC GL7^+^B220^+^ B cells (yellow). Enlarged regions from these sections indicate typical GC structures present within the follicles of WT and CD22^−/−^ mice, and detectable GL7^+^B220^+^ B cells within the follicles of CD154^TG^ mice, but not in CD154^TG^CD22^−/−^ mice (representative regions are shown for comparison). (A–C) Means significantly different from WT are indicated by asterisks (*p≤0.05, **p≤0.01), and between other indicated groups by crosses (†p<0.05, ††p<0.01). (**D**) Reduced GC B cells in CD154^TG^CD22^−/−^ mice as quantified by flow cytometry analysis. Mice were immunized with NP-CGG in alum, with spleens analyzed for GL7^+^B220^+^ B cells on day 10. Contour plots show mean (±SEM) GL7^+^ cell frequencies among total B220^+^ cells from *n* = 3 mice of each genotype. Bar graphs show mean (±SEM) GL7^+^ B cell numbers from naive (open bars) and immunized (filled bars) mice. In the contour plots, mean B10 cell frequencies significantly lower than those of WT mice are indicated by asterisks (**p≤0.01). In the bar graphs, means significantly different between naïve and immunized mice of the same genotype are indicated by asterisks (*p≤0.05); crosses for CD154^TG^CD22^−/−^ mice indicate that mean GL7^+^ cell numbers were significantly reduced relative to all other genotypes (††p<0.01).

Serum IgM concentrations were significantly higher in both CD22^−/−^ (155% increase) and CD154^TG^ (125% increase) mice at 4 mos of age when compared with WT mice, but were dramatically higher (1,220% increase) in CD154^TG^CD22^−/−^ mice (Figure 2A). At 12 mos of age, IgM levels were equally high in both CD154^TG^CD22^−/−^ and CD22^−/−^ mice. By contrast, serum IgG levels were reduced in both CD154^TG^CD22^−/−^ and CD22^−/−^ mice at 4 mos of age, and remained significantly reduced in 12 mo-old CD154^TG^CD22^−/−^ mice relative to all other genotypes. Thus, IgM levels were high in CD154^TG^CD22^−/−^ mice during early life, with little IgG Ab produced throughout life.

IgM autoAbs reactive with dsDNA, ssDNA and histone protein were significantly higher in both CD154^TG^CD22^−/−^ and CD22^−/−^ mice at 12 mos of age, while CD154^TG^ mice only had significant levels of IgM autoAbs reactive with dsDNA (Figure 2B). CD22^−/−^ mice also generated significant levels of IgG autoAbs reactive with dsDNA and ssDNA, but IgG autoAbs were virtually absent in CD154^TG^CD22^−/−^ mice. Therefore, CD154^TG^CD22^−/−^ mice produced IgM but not IgG autoAbs.

Following immunization with the TD Ag DNP-KLH in Freund's adjuvant, WT, CD22^−/−^, and CD154^TG^ mice generated significantly stronger DNP-specific IgG responses than CD154^TG^CD22^−/−^ mice (Figure 2C). Remarkably, classical GC structures were absent in the spleens of both CD154^TG^ and CD154^TG^CD22^−/−^ mice after the boost phase of DNP-KLH immunization as assessed by *in situ* immunofluorescence staining of frozen spleen sections (Figure 2C). Nevertheless, relatively rare GL7^+^B220^+^ B cells were detectable within the B cell follicles of spleens from CD154^TG^ mice, but not within the spleens of CD154^TG^CD22^−/−^ mice (Figure 2C, enlarged regions). Despite lacking classical GCs, the presence of detectable GL7^+^B220^+^ B cells in CD154^TG^ spleens (Figure 2C–D) coupled with their 4-fold spleen B cell expansion (Table 1) suggests that enough “GC-like” B cells are present to produce significant isotype switched IgG antibody. Similar results were obtained from mice immunized using the TD Ag NP_8_-CGG in alum, followed by quantitative analysis of total spleen GL7^+^B220^+^ B cells by flow cytometry. The frequency of spleen GL7^+^ B cells was significantly reduced in both CD154^TG^CD22^−/−^ (90% reduced) and CD154^TG^ (77% reduced) mice relative to WT mice after immunization (Figure 2D, contour plots). However, the total number of GL7^+^ B cells induced following immunization was specifically reduced in CD154^TG^CD22^−/−^ mice (62%, bar graphs), remaining normal in CD154^TG^ mice as a result of their expanded B cell pool. Thus, global IgG immune responses were specifically impaired in CD154^TG^CD22^−/−^ mice.

### B10 cells expand dramatically in CD154^TG^CD22^−/−^ mice

**Figure 3:**
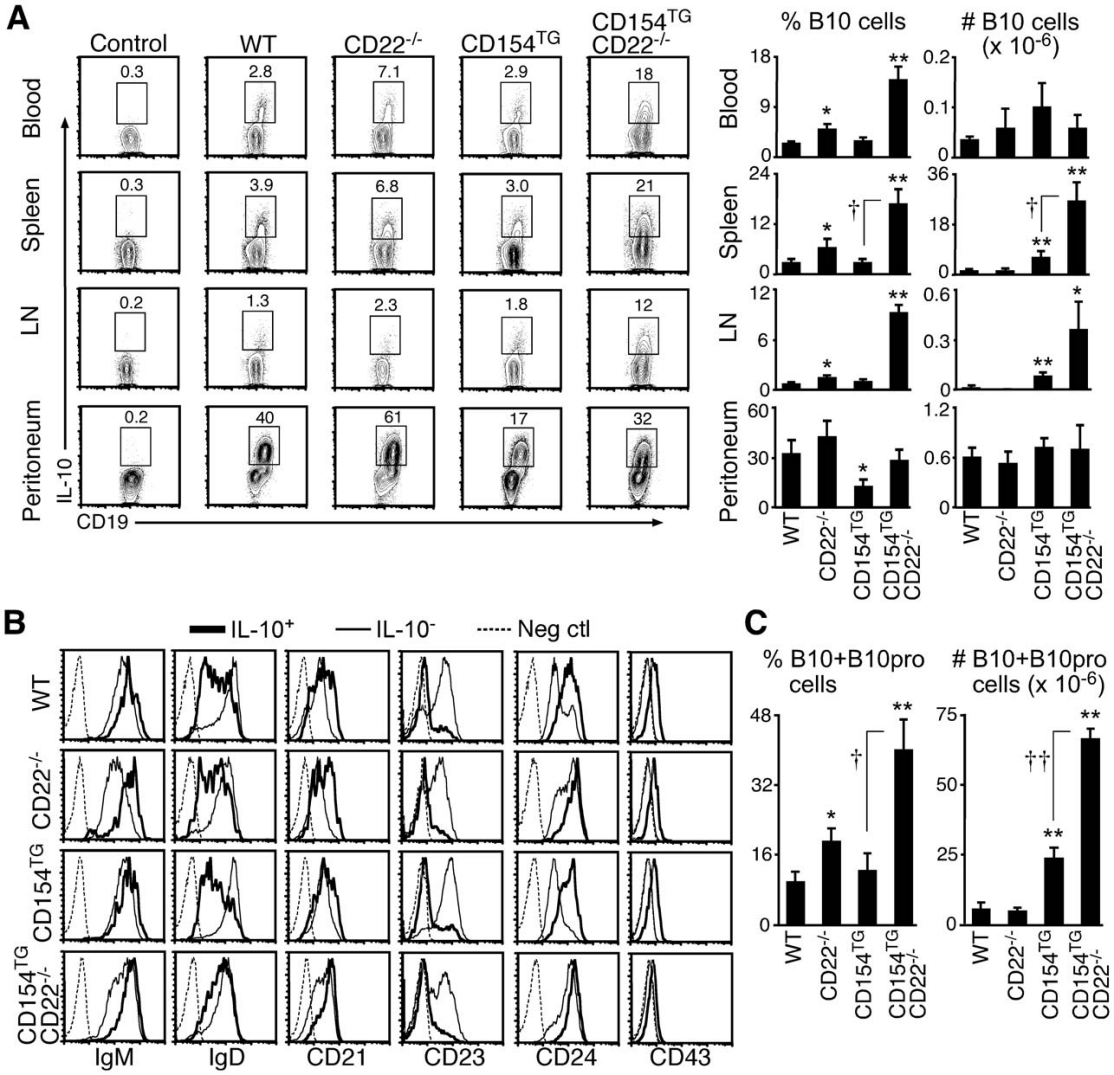
Spleen B10 and B10pro cells expand dramatically in CD154^TG^CD22^−/−^ mice. (**A**) Blood, spleen, inguinal LN, and peritoneal cavity B10 cell numbers in WT, CD22^−/−^, CD154^TG^, and CD154^TG^CD22^−/−^ mice. Leukocytes from the indicated tissues were cultured with LPS, PMA, and ionomycin for 5 h to induce IL-10 production, with monensin included in the cultures to block IL-10 secretion (L+PIM stimulation). The cells were then stained for cell surface CD19 and cytoplasmic IL-10 with flow cytometry analysis. Monensin treated cells served as negative controls for IL-10 expression. Representative contour plots gated on CD19^+^ cells are shown with the percentage of IL-10^+^ cells indicated. Bar graphs represent mean (±SEM) B10 cell frequencies (%) and numbers (#) from ≥3 mice of each genotype. (**B**) Similar B10 cell surface phenotypes among mouse genotypes. Splenocytes were cultured with L+PIM for 5 h and then stained for cell surface molecules and cytoplasmic IL-10. Histograms indicate cell surface molecule expression by IL-10^+^ (thick lines) and IL-10^−^ (thin lines) B cells. Dashed histograms represent isotype-matched control mAb staining. Results are representative of ≥3 mice of each genotype analyzed. (**C**) Spleen B10+B10pro cell frequencies and numbers. Splenocytes were cultured with an agonistic CD40 mAb, with L+PIM added during the final 5 h of 48 h cultures. Cytoplasmic IL-10^+^ B cells were identified as in (A). Bar graphs show means (±SEM) from ≥3 mice of each genotype. (A,C) Significant differences from WT mice are indicated: *p<0.05, **p<0.01. Means significantly different between CD154^TG^CD22^−/−^ and CD154^TG^ mice are indicated: †p<0.05, ††p<0.01.

Lending importance to the observed expansion of CD1d^hi^ B cells in CD154^TG^CD22^−/−^ mice (Figure 1D–E), a novel subset of regulatory IL-10-producing B cells termed ‘B10’ cells was recently identified within the spleen CD1d^hi^CD5^+^ subpopulation [27], [30], [36], [37]. Through *in vivo* adoptive transfer and depletion experiments, B10 cells were found to suppress autoimmunity and inflammation in mouse models of disease [30], [37], [38]. Remarkably, B10 cell frequencies and numbers were increased dramatically within the spleens of CD154^TG^CD22^−/−^ mice, with 16-, 15-, and 4.1-fold higher B10 cell numbers than in WT, CD22^−/−^, or CD154^TG^ mice, respectively (Figure 3A and Table 1). B10 cell numbers in tissues other than spleen were modest in all four genotypes (Figure 3A). B10 cells from WT, CD22^−/−^, CD154^TG^, and CD154^TG^CD22^−/−^ mice expressed similar phenotypes with augmented levels of CD19, IgM, CD21 and CD24, as well as low to intermediate levels of CD23 and IgD (Figure 3A–B). Despite their dramatic increase in spleen B10 cell numbers, serum IL-10 levels were not measurable in 4 mo-old CD154^TG^CD22^−/−^ mice (*n* = 9, data not shown). Enhanced *in vivo* survival signals alone were unlikely to explain B10 cell expansion, since spleen B10 cell frequencies and numbers were normal in 10 week-old Bcl-xL transgenic mice (2.4±0.9%, 9.2±0.9×10^5^, *n* = 6) relative to their WT littermates (2.6±0.2%, 7.4±1.4×10^5^, *n* = 3). Therefore, B10 cells were remarkably expanded in CD154^TG^CD22^−/−^ mice, while retaining a phenotype similar to WT B10 cells.

A fraction of spleen CD1d^hi^CD5^+^ B cells, called B10 progenitor (B10pro) cells, is induced to become IL-10 competent during 48 h cultures with agonistic CD40 mAb [27]. Since it is not yet possible to phenotypically distinguish existing B10 cells from *in vitro* matured B10pro cells, IL-10-producing B cells under these conditions are collectively defined as ‘B10+B10pro’ cells. Spleen B10+B10pro cell numbers in CD154^TG^CD22^−/−^ mice were 11-, 12-, and 2.8-fold higher than in WT, CD22^−/−^, and CD154^TG^ mice, respectively (Figure 3C and Table 1).

### B10 cells in CD154^TG^CD22^−/−^ mice are regulatory

**Figure 4:**
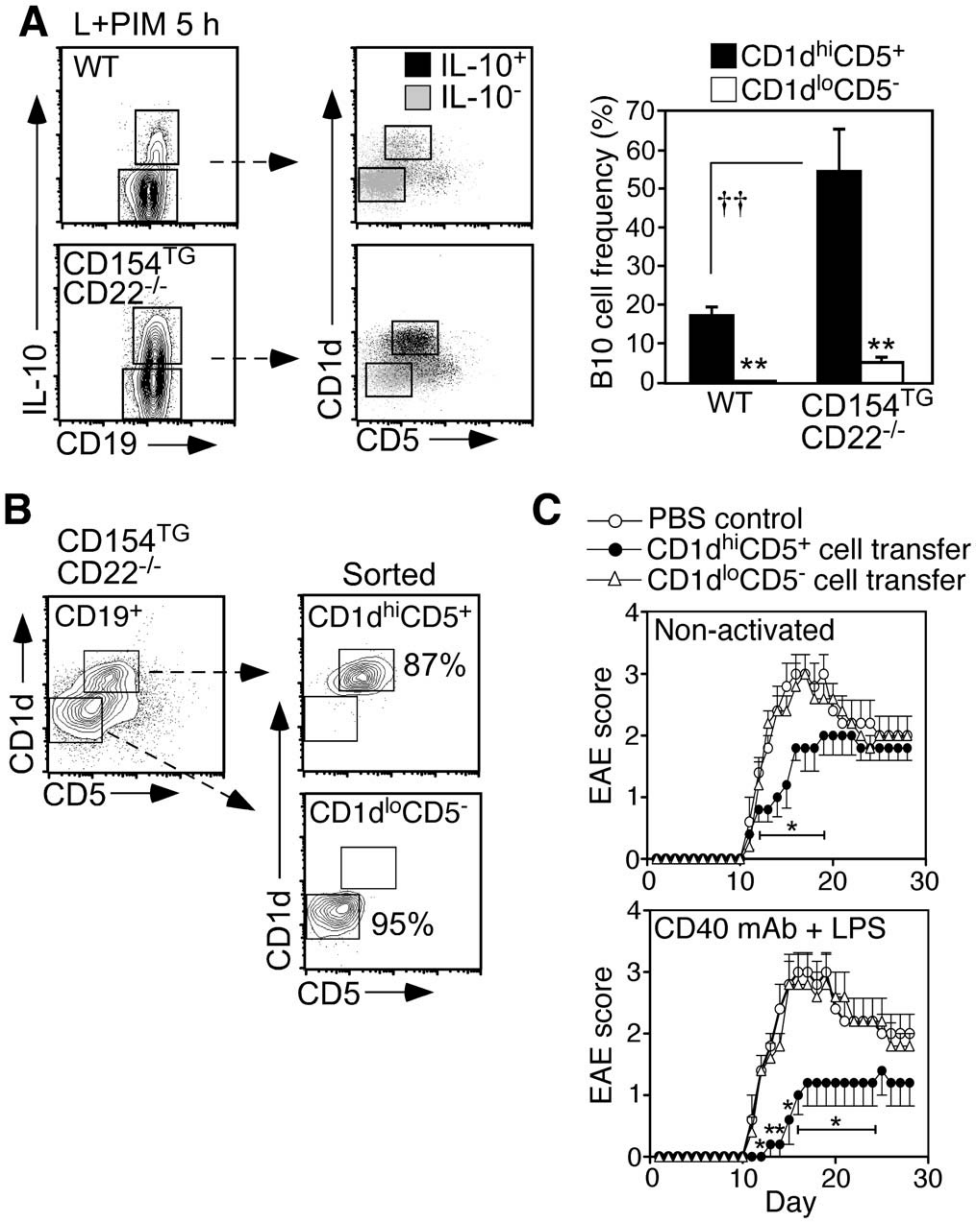
CD154^TG^CD22^−/−^ B10 cells are regulatory. (**A**) B10 cells in CD154^TG^CD22^−/−^ mice are found predominately within the spleen CD1d^hi^CD5^+^ B cell population. Splenocytes from WT and CD154^TG^CD22^−/−^ mice were cultured for 5 h with L+PIM to induce IL-10 expression, with the cells analyzed for cell surface CD19, CD1d and CD5, and intracellular IL-10 expression. CD19^+^IL-10^+^ and CD19^+^IL-10^−^ B cells (left panels, gated regions) were further gated to show relative CD1d and CD5 expression (merged dot plots, right panels). Bar graphs show the mean (±SEM) B10 cell frequencies within the indicated populations for ≥4 mice of each genotype based on the gated regions indicated in the merged dot plots. Mean B10 cell frequencies significantly different between cell populations from the same genotype are indicated by asterisks (**p≤0.01), and for the same population between genotypes by crosses (††p<0.01). (**B**) Analysis of CD154^TG^CD22^−/−^ spleen B cell purity within the CD1d^hi^CD5^+^ (B10-rich) and CD1d^lo^CD5^−^ populations following cell sorting of splenocytes stained for CD19, CD1d and CD5. These cells were subsequently used for the adoptive transfer experiments described in (C). (**C**) B10 cells from CD154^TG^CD22^−/−^ mice reduce EAE disease severity. Purified CD1d^hi^CD5^+^ or CD1d^lo^CD5^−^ spleen B cells from naïve CD154^TG^CD22^−/−^ mice (B) were either adoptively transferred into WT recipient mice immediately (non-activated) or were cultured with agonistic CD40 mAb for 48 h with LPS added during the final 5 h of culture before transfer. Other recipient mice received PBS alone (Control). One day after cell transfers, EAE was induced by MOG immunization. Values represent mean (±SEM) clinical EAE scores from 5 mice per group, with significant differences from PBS control mice indicated: *p<0.05.

Spleen B10 cells localize primarily within the CD1d^hi^CD5^+^ subpopulation in WT mice (Figure 4A) as described [37]. Likewise, spleen B10 cells in CD154^TG^CD22^−/−^ mice were found primarily within the CD1d^hi^CD5^+^ population (Figure 4A), where they represented >50% of the cells (bar graphs). This was >3 times the 15% frequency of B10 cells found within the CD1d^hi^CD5^+^ population of WT mice, and far above the 4% frequency of B10 cells found within the CD1d^lo^CD5^−^ subpopulation of CD154^TG^CD22^−/−^ mice.

The adoptive transfer of B10 cells from WT mice into syngeneic recipient mice significantly reduces the severity of EAE, an acutely-induced model of multiple sclerosis [30]. Whether B10 cells in CD154^TG^CD22^−/−^ mice were capable of influencing EAE was therefore assessed. Spleen CD1d^hi^CD5^+^ (B10-rich) B cells and CD1d^lo^CD5^−^ (B10-poor) B cells were purified from naive CD154^TG^CD22^−/−^ mice (Figure 4B) and adoptively transferred into WT mice 24 h before myelin oligodendrocyte glycoprotein (MOG) immunization to induce EAE. Unmanipulated CD1d^hi^CD5^+^ B cells from CD154^TG^CD22^−/−^ mice significantly reduced EAE severity (cumulative EAE score, 28±4, p<0.05), while disease was not altered in mice given CD1d^lo^CD5^−^ B cells (38±3) relative to mice given PBS as a control (40±5; Figure 4C, upper panel).

The frequency of IL-10-competent B10 cells among CD1d^hi^CD5^+^ B cells is enhanced by B10pro cell maturation *in vitro* (Figure 3) as described [27]. Therefore, CD1d^hi^CD5^+^ and CD1d^lo^CD5^−^ B cells purified from CD154^TG^CD22^−/−^ mice were stimulated with agonistic CD40 mAb for 48 h, with LPS added during the final 5 h of culture. The adoptive transfer of CD40/LPS-stimulated CD1d^hi^CD5^+^ B cells dramatically inhibited EAE progression (cumulative EAE score, 17±5, p<0.01), while CD1d^lo^CD5^−^ B cells did not (38±6; Figure 4C, lower panel). As such, B10 cells from CD154^TG^CD22^−/−^ mice had a regulatory capacity when transferred into WT mice.

### B10 cell depletion *in vivo* enhances IgG production in WT mice

**Figure 5:**
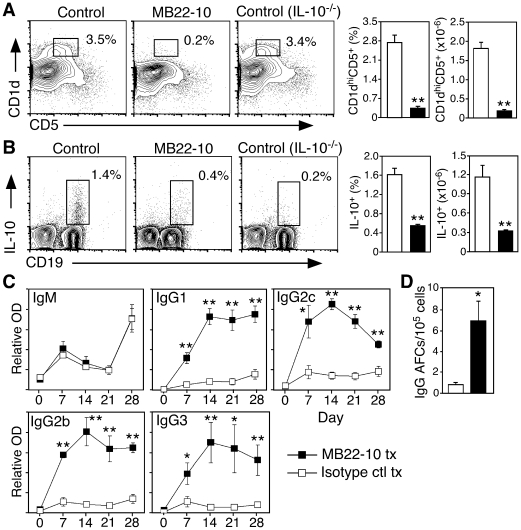
Endogenous B10 cells regulate IgG isotype switching in normal mice. (**A–B**) CD22 mAb treatment preferentially depletes CD1d^hi^CD5^+^ and B10 cells. Seven days after WT mice were given CD22 or control mAb, splenocytes were isolated and cultured in the presence of L+PIM for 5 h followed by cell surface CD19, CD1d and CD5, and intracellular IL-10 staining, with flow cytometry analysis. IL-10^−/−^ mice given control mAb were also evaluated for comparison. (A) CD22 mAb treatment preferentially depletes CD1d^hi^CD5^+^ B cells. Representative CD1d^hi^CD5^+^ B cell frequencies are indicated. (B) CD22 mAb treatment preferentially depletes B10 cells. Representative cytoplasmic IL-10^+^ B cell frequencies among spleen CD19^+^ cells are indicated. Bar graphs show mean (±SEM) frequencies and numbers of CD1d^hi^CD5^+^ B cells (A) or IL-10^+^ B10 cells (B) from control (open bars) and CD22 mAb treated (closed bars) mice. (**C–D**) B10 cells regulate IgG Ab responses. WT mice were given CD22 or control mAb (*n* = 3 per group) on day 0, and immunized with DNP-KLH without adjuvant on days 0 and 21. (C) Serum DNP-specific Abs were quantified by ELISA. (D) The frequency of B cells secreting DNP-specific IgG was determined by ELISPOT analysis of spleen cells harvested on day 28. (A–D) Significant differences between control and CD22 mAb-treated mice (≥3 mice for each treatment group) are indicated: *p<0.05; **p<0.01.

A mAb that engages the ligand binding domains of CD22 (MB22-10 mAb) preferentially depletes spleen B10 cells and B cells with a CD1d^hi^ phenotype in WT mice [31], [34]. Likewise, depletion of the CD1d^hi^CD5^+^ spleen B cell population that harbors most B10 cells was dramatic (>70%) in WT mice 7 days following a single dose of MB22-10 mAb relative to control mAb (Figure 5A). Spleen B10 cell frequencies and numbers were also reduced >60% in MB22-10 mAb-treated mice relative to control mAb treated mice (Figure 5B). Follicular CD1d^lo^CD5^−^ and other CD19^+^ non-B10 B cell frequencies and numbers were not significantly affected by MB22-10 mAb treatment (Figure 5A–B, and data not shown). Thus, CD22 mAb treatment efficiently depleted spleen B10 cells while leaving most other spleen B cells intact.

Effective B10 cell depletion by MB22-10 mAb in WT mice provides a system to verify that the expansion of B10 cells in CD154^TG^CD22^−/−^ mice contributes to their modest IgG responses. Since CD154^TG^CD22^−/−^ mice do not express CD22 and are thus not amenable to the use of CD22 mAb, it was determined whether B10 cell depletion by MB22-10 mAb would enhance IgG responses in WT mice immunized with a TD Ag in the absence of adjuvant. DNP-KLH immunization induced significant primary and secondary (day 21 boost) DNP-specific IgM responses in control mAb-treated WT mice, while DNP-specific IgG responses rose only slightly above background levels (Figure 5C). By contrast, MB22-10 mAb-treated mice generated normal Ag-specific IgM responses and robust DNP-specific IgG1, IgG2c, IgG2b, and IgG3 Ab responses that remained high. MB22-10 mAb treatment also significantly expanded the frequency of spleen IgG-secreting B cells in comparison with control mAb-treated mice (Figure 5D). Thereby, B10 cell depletion dramatically enhanced IgG immune responses in WT mice.

## Discussion

CD154 is expressed at relatively high levels by both T cells and B cells in SLE patients and in a mouse model of lupus, which is proposed to drive CD40 signaling, B cell hyperactivity, and autoAb production [16], [17], [18]. Although CD154 expression by B cells in the current study appears lower by comparison, the combination of CD154 expression and CD22 deficiency significantly enhanced B cell responses to CD40 signaling (Figure 1). B cell CD154 expression in CD154^TG^CD22^−/−^ mice also led to a remarkable 16-fold expansion of the regulatory B10 cell subset relative to WT mice. B10 cells normally represent only 1–3% of spleen B cells in WT mice [30], [37]. In fact, B10 cells alone represented 16% of spleen B cells in CD154^TG^CD22^−/−^ mice, while B10+B10pro cells represented 39% of spleen B cells. B10 cell expansion also paralleled a dramatic reduction in B cell isotype switching, with lower IgG Ab and autoAb levels in CD154^TG^CD22^−/−^ mice than were present in the parental mouse lines, even after immunization using a strong TD Ag in adjuvant. IgG deficiency was not observed in CD154^TG^ mice where spleen B10 cells were only expanded 3.8-fold and total spleen B cells were expanded 3.6-fold (Figure 3A and Table 1). Thus, heightened CD40 signaling through the combination of chronic CD154 expression and CD22-deficiency drove the *in vivo* expansion of regulatory B10 cells with functional activity, and limited the intensity of IgG immune responses, both of which may limit the pathogenic consequences of autoimmunity.

B cell negative regulation of immune responses through the production of IL-10 has been demonstrated in EAE [30], [39], [40], [41] and other mouse models of autoimmunity and inflammation [30], [36], [42], [43], [44], [45]. These B cells are called “B10 cells” because IL-10 secretion is required for their negative regulatory function, and other B cell subsets with their own unique regulatory properties may also exist [26]. B10 cells have a potent capacity to down-modulate immune responses during adoptive transfer experiments [30], [37], [38], [46], a property clearly retained by CD154^TG^CD22^−/−^ B10 cells (Figure 4C). Spleen B10 cells are predominantly contained within the phenotypically unique CD1d^hi^CD5^+^ subpopulation and are presumed to be functionally mature since they are competent to express quantifiable IL-10 after 5 h of *ex vivo* stimulation [30], [37]. B cell regulatory functions are also enhanced by *ex vivo* CD40 or LPS stimulation in inflammation and autoimmunity models [40], [47], [48], [49], although CD40 or TLR signaling are not required for B10pro or B10 cell generation [27]. However, CD40 signaling and LPS exposure can induce mouse B10pro cells to mature into functional IL-10-competent B10 cells [27], while LPS or CpG exposure can induce B10 cells to secrete IL-10. Thus, B10pro and B10 cells provide a fundamental linkage between the adaptive and innate immune systems. B10 cells in CD154^TG^CD22^−/−^ mice were phenotypically and functionally similar to B10 cells in WT mice, including their characteristic high levels of CD19 expression and ability to respond to LPS and CD40 signals (Figure 3; [37]). The current studies clearly demonstrate that enhanced B cell CD40 signaling *in vivo* leads to a remarkable phenotypic outcome characterized by B10 and B10pro cell expansion, with suppressed isotype switching and IgG autoAb production that may be attributable to the regulatory activity of these cells through their production of IL-10.

Depleting the relatively small number of endogenous B10 cells in wild type mice significantly enhanced isotype switching and IgG production in response to immunization in the absence of adjuvant (Figure 5C–D). B10 cell depletion by CD22 mAb also enhances cellular immunity during EAE initiation [31]. By contrast, expanded B10 cell numbers in CD154^TG^CD22^−/−^ mice paralleled their IgG-deficient phenotype. Further supporting a mechanistic explanation for reduced IgG production in CD154^TG^CD22^−/−^ mice, B10 cells from CD154^TG^CD22^−/−^ mice retained their regulatory function and suppressed EAE during adoptive transfer experiments (Figure 4C). It was not possible to directly compare the functional activities of B10 cells from WT and CD154^TG^CD22^−/−^ mice *in vivo* because of their different relative frequencies within the CD1d^hi^CD5^+^ B cell subsets used for adoptive transfer experiments (Figure 4A), the fact that it is not possible to quantify Ag-specific B10 cell frequencies, and that B10 cells in CD154^TG^CD22^−/−^ mice are not amenable to CD22 mAb-induced depletion. Nonetheless, the regulatory activities of CD1d^hi^CD5^+^ B cells from CD154^TG^CD22^−/−^ mice were strikingly similar to those published for CD1d^hi^CD5^+^ B cells from WT mice [31], and B10 cells from CD154^TG^CD22^−/−^ and WT mice produced similar levels of cytoplasmic IL-10 following *ex vivo* stimulation. Globally impaired IgG responses were also unique to CD154^TG^CD22^−/−^ mice, arguing against constitutive CD40 signaling or continuous CD154 internalization, as also occurs in CD154^TG^ mice (Figure 1A,F), as the explanation for this observation. It is also unlikely that B10 cells inhibited B cell differentiation directly since serum IgM levels were elevated in CD154^TG^CD22^−/−^ mice. Thus, B10 cell depletion in WT mice and expansion in CD154^TG^CD22^−/−^ mice had the predicted biologic effects of facilitating and inhibiting, respectively, IgG immune responses.

Agonistic CD40 mAb treatment *in vivo* “short-circuits” humoral immunity and impairs IgG production, GC formation, and B cell memory [13]. Tsubata and colleagues have also found accelerated termination of GC reactions to T cell-dependent Ags in hemizygous CD154^TG^ mice, although IgG production remained robust [14], [15]. CD40 agonists also induce extrafollicular B cell differentiation. The “short circuiting” of IgG responses described above under robust CD40 ligation conditions (e.g., agonistic CD40 mAb) may be explained in part by the expansion and/or maturation of B10 and B10pro cells, as occurred in CD154^TG^CD22^−/−^ mice. Consistent with this, B10 cells proliferate more rapidly than non-B10 cells following mitogen stimulation [27]. Therefore, the rapid expansion of Ag-specific B10 cells within extrafollicular foci of CD154^TG^CD22^−/−^ mice may result in Ag consumption and/or clearance by IgM before the initiation of germinal center reactions and IgG production. Alternatively, B10 cells may indirectly regulate germinal center formation by altering TD immunity or the ability of dendritic cells to act as Ag-presenting cells during T cell activation [31], which are required for efficient B cell isotype switching.

Treatment of WT mice with the MB22-10 mAb that blocks CD22 ligand binding preferentially depleted spleen B10 and CD1d^hi^CD5^+^ B cells, but not follicular B cells (Figure 5A,B) as described [31]. While CD22 ligand binding is needed for the optimal survival of some B cell populations, such as MZ B cells [34], [35], [50], B10 cell development was normal in CD22^−/−^ mice and dramatically expanded in CD154^TG^CD22^−/−^ mice. As such, CD22 ligation with this particular mAb may induce signals that promote B10 cell apoptosis, alter their maturation, and/or affect their tissue distribution. B10 cell localization may be critical for function since spleen CD1d^hi^B220^+^ B cells were identified in both the MZ and follicular regions (Figure 1D), suggesting that B10 cells inhabit both sites. In support of this, spleen MZs were undetectable in CD22^−/−^ mice, yet these mice had significant numbers of B10 and CD1d^hi^B220^+^ B cells scattered throughout their follicles. Intrafollicular CD1d^hi^ B cells were especially prominent in CD154^TG^CD22^−/−^ mice (Figure 1D). In any case, B cell depletion by the MB22-10 mAb is not dependent on Ab-dependent cellular cytotoxicity or complement activation [34]. In addition, the MB22-10 mAb impairs malignant B cell survival in mice [34]. Therefore, therapeutic B10 cell depletion may be useful for enhancing IgG responses, or for the treatment of tumors and immunosuppression.

Foxp3^+^CD25^+^ Treg cell numbers were normal in both CD154^TG^ and CD154^TG^CD22^−/−^ mice (Table 1). Similarly, B10 cells do not appear to directly regulate Treg cell numbers in an EAE model, where these two regulatory subsets function independently [31]. This suggests that the potential suppressive effect of B10 cells on IgG production did not result from Treg cell expansion. By contrast, the adoptive transfer of CD1d^hi^CD5^+^ B cells from wild type mice into CD19-deficient NZB/W F1 mice leads to a significant increase in Treg numbers [46]. While these results suggest that B10 cells could drive Treg cell expansion, the majority of spleen CD1d^hi^CD5^+^ B cells are not B10pro or B10 cells [27]. Thus, the adoptive transfer of non-B10 cells may independently promote CD4^+^ Treg cell generation. This possible explanation may also apply to studies suggesting that Breg cell-induced inhibition of inflammation is partly driven by Breg cell-induced Treg cell expansion. Nonetheless, spleen IL-10-competent T cell numbers were significantly increased in both CD154^TG^ and CD154^TG^CD22^−/−^ mice relative to WT and CD22^−/−^ mice, in parallel with increased CD4^+^ and CD8^+^ memory T cell numbers (Table 1). The increase in IL-10-competent and memory T cells may therefore result from B cell CD154 expression. It is unlikely that the increase in IL-10-competent and memory T cells drives B10 cell expansion since B10 cell expansion was limited to CD154^TG^CD22^−/−^ mice. Thus, B cell expression of CD154 appears to have intrinsic effects on B cells as well as extrinsic effects on cellular immunity.

T and B cell CD154 expression in SLE patients is proposed to drive autoimmunity [16], [17], [18], and a clear role for aberrant CD40 signaling in other inflammatory autoimmune diseases has emerged in recent years [51], [52]. It has also been suggested that chronic CD40 signaling, particularly in patients with SLE, functionally inactivates a subset of regulatory B cells [53]. However, the current studies suggest that B cell CD154 expression may predispose lupus patients towards enhanced B10 cell production. Consistent with this, B10pro cell numbers are expanded in SLE patients, and can be induced to mature and acquire IL-10 competence following agonistic CD40 stimulation [28]. Therefore, constitutive, robust CD40 signals may drive functional B10pro cell expansion and limit the pathogenic consequences of autoimmunity by reducing Ab isotype switching. Moreover, this study predicts that certain combinations of genetic traits that can individually induce autoimmune disease may actually reduce autoimmunity by expanding B10 cell numbers. For example, CD22^−/−^ mice produce high-affinity serum IgG autoAbs spontaneously with age [54], and hemizygous CD154^TG^ mice can develop relatively mild autoimmunity [19], [20]. B10 cell numbers are also expanded in type 1 diabetes-prone NOD mice, and in lupus-prone MRL*lpr* and NZB/W F_1_ mice, even prior to the appearance of disease [27], [38]. Similarly, blood B10pro cell numbers are expanded significantly in humans with lupus and other autoimmune diseases [28]. The observation that manipulation of the CD40 and CD22 signaling pathways can drive resident B10 cell expansion *in vivo* also highlights a potential therapeutic benefit for expanding B10 cells in humans to limit the pathogenic consequences of humoral IgG responses to self Ags.
